# Unusual syphilis manifestation: From breast implant rupture to systemic erythroderma

**DOI:** 10.1016/j.idcr.2025.e02170

**Published:** 2025-02-01

**Authors:** Vivien Moris, Eliott Laine, Sahal Khan, Leslie Ann See

**Affiliations:** a Department of Plastic and Reconstructive Surgery, University Hospital of Dijon, France; b Division of Orthopaedic Surgery and Musculoskeletal Trauma Care, University Hospital of Dijon-Burgundy, Dijon, France

**Keywords:** Syphilis, Breast implant complication, Transgender health, Erythroderm, Lymphadenopathy

## Abstract

This case report describes a rare and unusual manifestation of syphilis in a 55-year-old transgender female with a history of breast implant surgery. The patient presented with a painless enlargement of the right breast, accompanied by pruritus and skin lesions. Initial misdiagnosis led to treatment for scabies, which worsened into diffuse erythroderma. Further examination revealed breast implant rupture and systemic lymphadenopathy. Surgical removal of the implant and seroma drainage was performed. Pathological findings showed chronic inflammation, with concerns about a cutaneous T-cell lymphoproliferative disorder, but no signs of malignancy were observed. Serological tests confirmed active syphilis with a positive TPHA titer and VDRL result. This case highlights the importance of considering infectious etiologies, such as syphilis, even in patients with atypical clinical presentations, including those involving breast implants and skin conditions. Timely diagnosis and appropriate management are critical to prevent systemic complications in similar cases.

A 55-year-old transgender female (male-to-female) patient with a history of breast implant surgery in 1994 presented to the emergency department with a painless, progressive enlargement of the right breast over two weeks. The patient was afebrile and did not report weight loss, but experienced fatigue for the previous 15 days.

Concurrent with breast swelling, the patient developed pruritus and scattered skin lesions. Initially misdiagnosed as scabies, treatment with ivermectin, topical permethrin, and antihistamines led to diffuse erythroderma with desquamation affecting 90 % of the body. Bilateral mobile axillary and inguinal lymphadenopathy were noted on physical examination.

Initial blood tests showed hemoglobin at 14.5 g/dL, leukocytes at 9 G/L, and eosinophilia at 1.69 G/L, with normal electrolytes, creatinine, and liver function tests. C-reactive protein was elevated at 21 mg/L. A contrast-enhanced thoracic CT scan revealed extracapsular rupture of the right breast implant, a 22 × 19 cm peri-implant seroma, and bilateral axillary lymphadenopathy, initially suspected to be silicone-related. No pectoral abscess or pulmonary infection was detected.

Surgical removal of the implant and capsulotomy yielded 1.5 liters of stercoral fluid. Pathology from the breast capsule indicated chronic inflammation without signs of anaplastic large-cell lymphoma. An excised inguinal lymph node showed hyperplasia of interdigitating dendritic cells, suggestive of dermatopathic lymphadenitis. Skin biopsy revealed chronic inflammation, raising concerns for a cutaneous T-cell lymphoproliferative disorder.

Serological testing ultimately confirmed active syphilis infection with a TPHA titer of 1/1280 and VDRL positive at 1/4.

**Figure:**
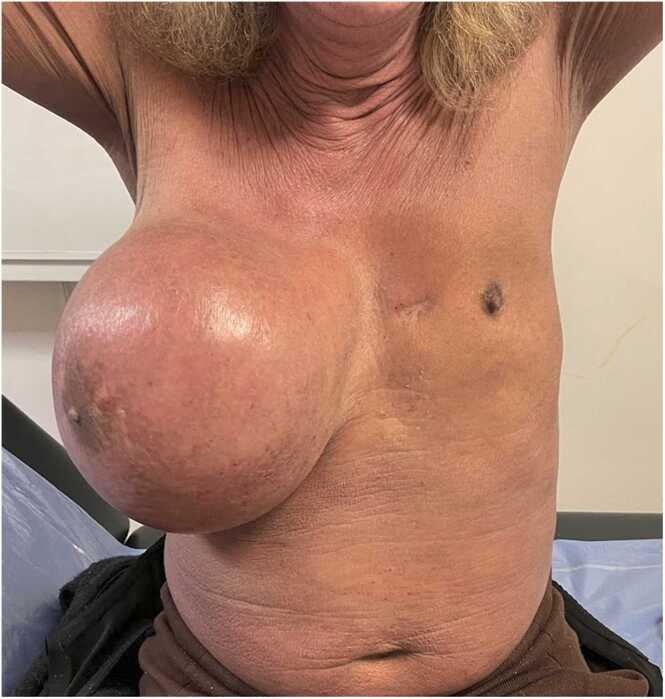

## Conclusion

This case illustrates an active syphilis infection, initially presenting with right breast implant involvement, progressing to systemic erythroderma and lymphadenopathy.

## CRediT authorship contribution statement

**moris vivien:** Conceptualization, Data curation, Validation, Writing – original draft, Writing – review & editing. **Laine Eliott:** Data curation, Methodology, Resources. **Khan Sahal:** Investigation, Methodology, Supervision. **See Leslie Ann:** Validation, Writing – review & editing.

## Declaration of Competing Interest

The authors declare that they have no known competing financial interests or personal relationships that could have appeared to influence the work reported in this paper.

